# Statin and Bisphosphonate Induce Starvation in Fast-Growing Cancer Cell Lines

**DOI:** 10.3390/ijms18091982

**Published:** 2017-09-15

**Authors:** Heidrun Karlic, Florian Haider, Roman Thaler, Silvia Spitzer, Klaus Klaushofer, Franz Varga

**Affiliations:** 1 Ludwig Boltzmann Cluster Oncology, Hanusch Hospital, Vienna 1140, Austria; heidrun.karlic@meduniwien.ac.at; 2 Ludwig Boltzmann Institute of Osteology at the Hanusch Hospital of WGKK and AUVA Trauma Centre Meidling, 1st Medical Department, Hanusch Hospital, Vienna 1140, Austria; florian.haider@students.boku.ac.at (F.H.); silvia.spitzer@osteologie.at (S.S.); klaus.klaushofer@osteologie.at (K.K.); 3 Departments of Orthopedic Surgery and Biochemistry and Molecular Biology, Mayo Clinic, Rochester, MN 55905, USA; Thaler.Roman@mayo.edu

**Keywords:** statin, bisphosphonate, cancer cell lines, starvation

## Abstract

Statins and bisphosphonates are increasingly recognized as anti-cancer drugs, especially because of their cholesterol-lowering properties. However, these drugs act differently on various types of cancers. Thus, the aim of this study was to compare the effects of statins and bisphosphonates on the metabolism (NADP^+^/NADPH-relation) of highly proliferative tumor cell lines from different origins (PC-3 prostate carcinoma, MDA-MB-231 breast cancer, U-2 OS osteosarcoma) versus cells with a slower proliferation rate like MG-63 osteosarcoma cells. Global gene expression analysis revealed that after 6 days of treatment with pharmacologic doses of the statin simvastatin and of the bisphosphonate ibandronate, simvastatin regulated more than twice as many genes as ibandronate, including many genes associated with cell cycle progression. Upregulation of starvation-markers and a reduction of metabolism and associated NADPH production, an increase in autophagy, and a concomitant downregulation of H3K27 methylation was most significant in the fast-growing cancer cell lines. This study provides possible explanations for clinical observations indicating a higher sensitivity of rapidly proliferating tumors to statins and bisphosphonates.

## 1. Introduction

As previously reported [1], evidence from both in vitro and in vivo data has demonstrated that drugs such as statins and bisphosphonates targeting the mevalonic acid pathway and consequently the synthesis of isoprenoids and cholesterol exert, beyond their lipid-lowering effects, pleiotropic actions, including immune regulation [1,2] and cancer prevention [3,4] as well as epigenetic effects [5]. However, observed differences in the anti-cancer potency of these drugs might be related to cell type specific inhibitory activities from these drugs on uptake of glucose and other nutrients such as essential amino acids [6,7,8,9].

The anti-tumorigenic effects of statins vary between different types of cancer: the amelioration of breast cancer prognosis was extensively reviewed [10]; survival or recurrence by statin was documented in one study with 146,326 participants [11] and other studies with 75,684 [12] or 124,669 [13] women. There is also data available on the beneficial effect of bisphosphonates for the treatment of breast cancer [14]. The curative effect of bisphosphonates on breast cancer is also mentioned in recent publications [15] discussing potential options for the treatment of lysyl oxidase positive, estrogen receptor negative (LOX+, ER−) breast cancer patients. In prostate cancer patients, a statin-associated reduction of mortality has been documented in more than 100,000 cases [16]. However, the treatment success also appears to be influenced by mitochondrial DNA mutations and associated metabolic consequences [17] and non-responders to statin-therapy with persistent high serum cholesterol still have a higher cancer risk [18].

Epidemiological evidence projecting statins and/or bisphosphonates as anticancer agents is conflicting, which largely depends on the type of cancer in question [19,20] and, to the best of our knowledge, no epidemiological data exist on the application of these drugs in osteosarcomas. Based on the working hypothesis, that statins and bisphosphonate-responses could be linked with the basic proliferation rate of respective tumor cells, the aim of this study was to elucidate underlying mechanisms by combining metabolic analyses with transcriptomic and complementary immune blot analyses.

## 2. Results

### 2.1. Cell Cycle

Mevalonate-deprivation related cell-cycle arrest and cell quiescence was first published more than 25 years ago [21]. A quiescence marker resulting from this pioneering study is the downregulation of the DNA polymerase A1 (*POLA1*), which we also found downregulated in our study (Table 1).

**Figure 1:**
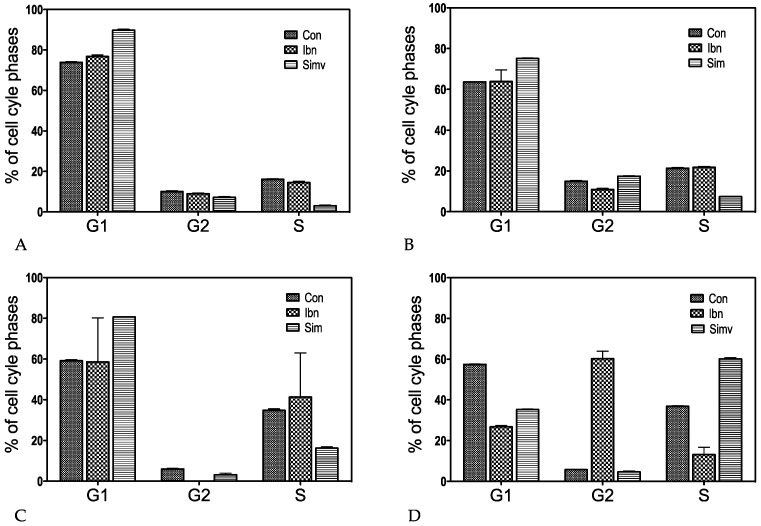
The distribution of cell cycle phases was analyzed by flow cytometry in PC-3 prostate cancer (**A**); MDA-MB-231 breast cancer (**B**); U-2 osteosarcoma (**C**) and MG-63 osteoblast-like (**D**) cells.

This is confirmed by the common feature of seven best-regulated genes, which showed a more than 5-fold reduction in at least two cell lines (upregulation upregulation of sestrin 2 *SESN2* and downregulation of topoisomerase 2A *TOP2A*, thymidilate synthase *TYMS*, anillin actin binding protein *ANLN*, *SESN2*, DNA damage inducible transcript 4 *DDIT4*, and cyclin A2 *CCNA2*, cyclin B1 *CCNB1* referred to a role in regulation of “cell cycle”). (Supplementary Table S1: Amount of “PubMed”—results with the seven best-regulated genes plus “cell cycle”). Based on these results, we concluded that in tumor cells statins as well as bisphosphonates primarily induce cell cycle arrest (Figure 1). Indeed, simvastatin induced cell cycle arrest in G1 in PC-3 prostate carcinoma, MDA-MB-231 breast cancer, and U-2 osteosarcoma (OS) cells (Figure 1A–C), whereas in MG-63 osteoblast-like cells cell cycle arrest was increasingly observed in the S-phase. Furthermore, MG-63 was the only cell line where an ibandronate-induced enrichment in the G2-phase could be observed (Figure 1D).

In agreement with the cell-cycle effects, a remarkable reduction in the mRNA expression of the S-phase associated cyclins *CCNA2* and *CCNB1* (Table 2 and Table 3) was observed, thus confirming previous results with atorvastatin [22].

The stem cell-related forkhead box M1 *FOXM1* gene, which is known for its activation in the G2/M phase [23,24], is significantly downregulated in ibandronate-treated as well as in simvastatin-treated PC-3 and MDA-MB-231 cells. In MG-63 and U-2 OS cells, this regulation was less prominent, probably because simvastatin induced an S-phase arrest and ibandronate induced rather a G2 arrest in MG-63 cells and an S-phase arrest in U-2 OS cells, despite a G1 arrest upon simvastatin treatment in this cell line (Table 4).

This observation could be partially explained by the fact that the retinoblastoma associated RB transcriptional corepressor 1 (RB1), which plays a critical role for the exit from the G1 to the S-phase, is mutated in PC-3 [25] and MDA-MB-231 cells [26].

### 2.2. Influence of Statins and Bisphosphonates on NADP(H) Production

NADPH is involved in many metabolic reactions. Statins act as inhibitors of the HMG-CoA reductase, which is itself a NAD(P)H dependent enzyme. As we have shown before, simvastatin and ibandronate downregulated the proliferation of epithelial and bone related mesenchymal cancer cells [5]. Building on these results, we were interested if these drugs influence the cellular levels of NADPH and NADP^+^ or the ratio between these two metabolites. Therefore, we measured the concentrations of NADPH and NADP^+^ as well as the ratio between NADPH/NADP^+^ (Table 5).

The weaker metabolic effect of ibandronate as compared to simvastatin in all cell lines except MG-63 could be attributed to the number of genes regulated by this bisphosphonate (Table 6).

**Figure 2:**
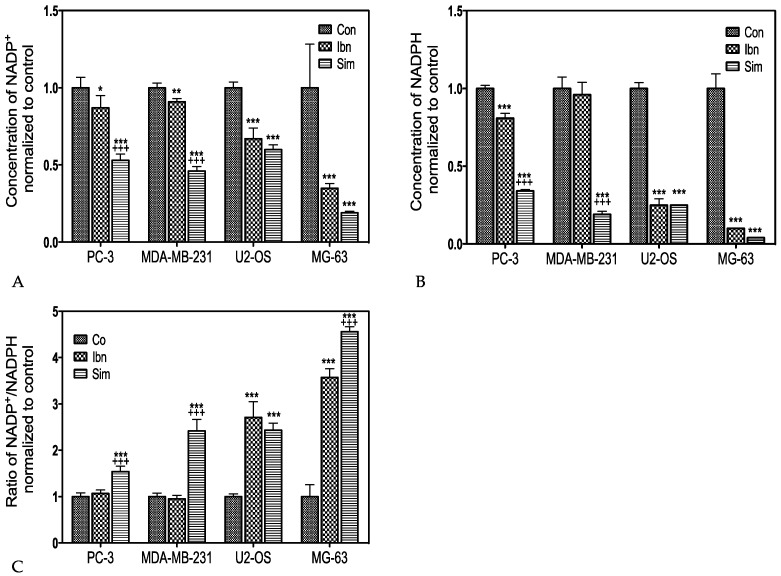
Effect of statin and bisphosphonate on NADP(H) production. Cell lines were treated with EC50-associated concentrations of simvastatin (Sim, 1 µM for PC3, 0.5 µM for MDA-MB-231, 3 µM for U-2 OS, and 10 µM for MG-63) or ibandronate (Ibn, 50 µM for all cell lines) for 72 h. Then, concentrations of NADP^+^ (**A**) and NADPH (**B**) were analyzed using the NADP/NADPH Glo Assay (Promega) and the NADPH/ NADP^+^ ratio was calculated (**C**). The “fold”–ratio is given on the *y*-axis. Bars represent the mean ± SD; * *p* < 0.05, ** *p* < 0.01, *** *p* < 0.001, control (Con) vs. treatment; † *p* < 0.05, †† *p* < 0.01, ††† *p* < 0.001, Ibn vs. Sim; *n* = 4.

The effects of ibandronate and simvastatin on NADP^+^ and NADPH levels in highly proliferative tumor cells like the epithelial PC-3 and MDA-MB-231 cell-lines as well as the osteosarcoma U-2 OS cell-line, and in slower proliferating MG-63 osteosarcoma cells after treatment for 72 hours is shown in Figure 2.

In the epithelial tumor cells PC-3 and MDA-MB231, NADP^+^ (Figure 2A) and NADPH (Figure 2B) concentrations decreased significantly after treatment with simvastatin. However, the effect was clearly milder after exposure to ibandronate. Of note, there was no significant difference in treatment response between simvastatin and ibandronate in the osteosarcoma cell lines showing a general reduction after 72 h. In the drug responsive cell-lines both, NADP^+^ and NADPH concentrations were decreased by more than 60%. Simvastatin significantly increased the NADP^+^/NADPH ratio in the fast-growing epithelial derived cell lines (Figure 2C) and both drugs in the bone related mesenchymal cell lines, thus confirming a direct antimetabolic effect of bisphosphonates in mesenchymal cell lines such as U-2 OS [31]. Recent data on the influence of cell cycle regulators (and their stimulation by oncogenes such as RAS) on metabolism [32,33] underline a close connection between energy metabolism and proliferation. However, the role of enzymes such as NADPH oxidase NOX4 (Table 7) and NOS (Table 8) in regulating the equilibrium between NADPH and NADP^+^ is rather related to the production of reactive oxygen species in resting cells.

Although the specific regulation of the NADPH-oxidase *NOX4* (Table 7), which is known for its role in the production of reactive oxygen species in mesenchymal cells, could provide some explanation, there are still open questions relating to the relatively low basal expression of *NOX4* (minus 50% as compared to the cell cycle genes mentioned in Figure 1A–D) in U-2 OS cells down to 31%.

However, there is a reciprocal relationship of NOX4 with endothelial nitric oxide synthase NOS1, which showed a 3-fold increase in simvastatin-treated U-2 OS cells (Table 8). Considering an association with uncoupling and matrix protein expression, which includes a role of sestrin 2 [34], this could provide a link towards the above-mentioned impairment of glucose metabolism.

Prenyl (decaprenyl) diphosphate synthase subunit 1 *PDSS1*, a critical enzyme for the synthesis of coenzyme Q [35] and for the respective NADPH-producing respiratory chain pathway, was downregulated in all treated cell lines (Table 9).

### 2.3. Autophagy

Autophagy is known to act as a temporary survival mechanism in response to stress-induced damage of the endoplasmic reticulum (ER) and/or nutrient starvation [36].

**Figure 3:**
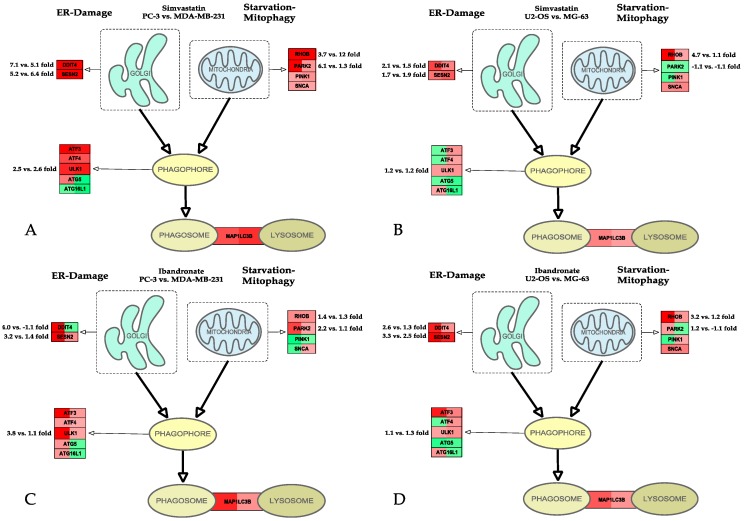
Results of transcriptomic analyses of autophagy-associated genes from PC-3 (**A**); MDA-MB-231 (**B**); U-2 OS (**C**) and MG-63 (**D**) cells using the Pathvisio tool. Upregulated genes are red, downregulated genes are green. ER, endoplasmic reticulum.

The respective gene network, which is responsible for phagosome-formation and mitophagy, has been analyzed (Figure 3). Being key molecules in the autophagy-signaling cascade, DDIT4 and SESN2 signal damage of the ER whereas nutrient starvation is accompanied by stimulation of ras homolog family member B RHOB, which initiates a reduction of energy-consuming mitochondria (mitophagy) and recycling processes of organelles, which are lysed during autophagy. *DDIT4* and *SESN2* were significantly upregulated by simvastatin and ibandronate in all treated cell lines, except for the MDA-MB-231 cells, where *DDIT4* was not upregulated by ibandronate. SESN2 is also known for its antioxidative function and it promotes cell survival by downregulating apoptosis and increasing autophagy via inhibition of mTOR signaling [37]. A coincidence with stimulation of *RHOB*, which is known to be upregulated by nutrient shortage, could indicate an increase in protein degradation and recycling through an endolysosomal pathway [38], especially in simvastatin-treated PC-3 and MDA-MB-231 cells (Figure 3A).

We also detected a concurrent upregulation of phagosome-associated markers such as the autophagy initiating kinase *ULK1* (unc-51 like autophagy activating kinase 1, also known as ATG1), which was upregulated in all investigated cell lines (Figure 3). ULK1 plays a key role in an autophagy-associated protein complex, which is under control of mTOR [39,40]. A similar pattern of upregulation was found for LC3 (also known as microtubule associated protein 1 light chain 3 alpha, MAP1LC3B), which is responsible for the autophagosome-lysosome-fusion. LC3 is also upregulated by inhibitors of the histone methylase EZH2 [41]. EZH2 is increasingly recognized as a target for the treatment of various neoplastic diseases, especially those with RAS-mutations [42,43,44]. Considering the fact that 3-hydroxy-3-methylglutaryl-CoA (HMGCR) reductase is a direct target of statins and is immediately situated at the endoplasmic reticulum, it appears possible that inhibition of HMGCR causes ER-stress which is known to cause autophagy [41].

Interestingly, the mitophagy marker PARK2 [45] is upregulated in the cell lines with epithelial background and weakly in the fast-growing mesenchymal U-2 OS cell line, suggesting mitophagy in these cells.

Only some genes of the autophagy-associated ATG–family were significantly regulated (Figure 3A–D), but the high basal expression (7% to 17% of the 18S ribosomal gene) of some genes of this family suggests that the abundant expression of these factors would be sufficient to support non-canonical autophagy.

Considering the regulatory influence of microRNAs on autophagy [46], we checked the expression levels of microRNAs, where the extent of regulation is associated with cell type. Table 10 shows the regulation of *MIR21*, but a significant stimulation was only observable in ibandronate-treated U-2 OS cells.

A minus 3-fold (=minus 70%) ibandronate-induced downregulation of *MIR21* in the U-2 OS osteosarcoma cells might be associated with the known drug-induced RAS-inactivation in this cell line [47].

### 2.4. Histone Demethylation as a Sign of Starvation

Recently, it has been demonstrated that statins downregulate the histone methylase EZH2 [48] and promote autophagy as a result of disturbed uptake of the amino acid methionine [41]. Downregulation or inhibition of EZH2 decreases histone-3 methylation on lysine 27 and could be associated with the downregulated cell cycle activity (see also Figure 1) [49,50]. The genome wide expression analysis in Table 11 shows that treatment of PC-3 and MDA-MB-231 cells resulted in a more prominent downregulation of this demethylase by simvastatin, while ibandronate only weakly influenced their expression but no regulation was suggested in both other cell lines.

**Figure 4:**
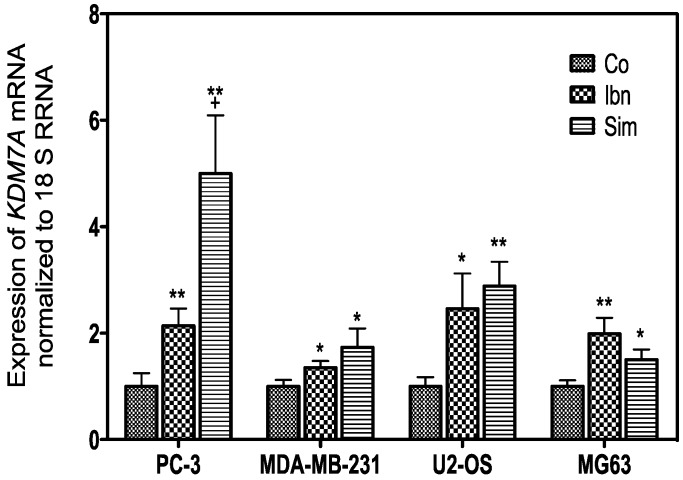
Effect of ibandronate (Ibn) and simvastatin (Sim) on the mRNA expression of the histone demethylase *KDM7A*. Bars represent the mean ± SD; * *p* ≤ 0.05, ** *p* ≤ 0.01, Control (Co) vs. treatment. † *p* ≤ 0.05, ibandronate vs. simvastatin; *n* = 3.

Although the expression of the H3K27me3 methylase *EZH2* is only moderately suppressed by simvastatin or ibandronate, a general upregulation of the histone H3 lysine 9 and histone H3 lysine 27 demethylase *KDM7A* (also known as *JHDM1D*, Table 12), which is known to be induced during nutrient starvation, was observed [51]. This indicates a reduction of H3K27me3 methylation after inhibition of the mevalonate pathway in the analyzed cell lines. This confirms the possible association with starvation and autophagy, because similar observations were reported to be associated with hypoxia [52]. Quantitative reverse transcription real time polymerase chain reaction (RT-qPCR) confirmed the GeneChip data as shown in Figure 4. Moreover, we could confirm a stronger stimulation in PC-3 and U-2 OS and a weaker regulation in MDA-MB-231 and MG-63 cells.

**Figure 5:**
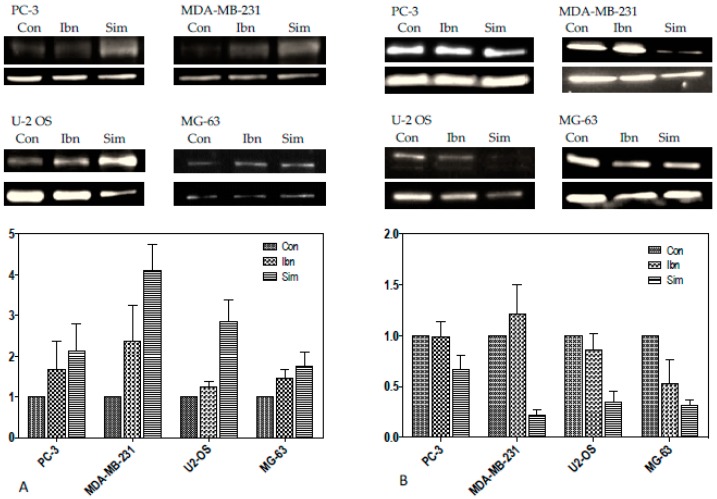
The effect of a simvastatin and ibandronate (treatment for 6 days with indicated EC50 concentrations as shown in Table 5) on the protein expression of the histone demethylase KDM7A is shown. The photographs show KDM7A (95 kD) in the upper lanes and the actin ACTA1 (42 kD) in the lower lanes for comparisons. “Con” refers to untreated control cells, “Ibn” to ibandronate-treated cells, and “Sim” to simvastatin-treated cells. Chemo-luminescence for the analysis of protein bands was measured with an image acquisition. Results of chemiluminescence measurements are given as means of three immunoblots.

Immunoblots confirmed a regulation of the histone demethylase on the protein level (Figure 5A). The Jumonji histone demethylases (Jmj-KDM) belong to an important class of transcriptional coactivators because they erase the repressive marks H3K9me2/1, H3K27me2/1, and H4K20 me1. Some members of this family were identified to play a tumor-suppressive role through the reinforcement of TP53-driven growth arrest and apoptosis [53], thus representing therapeutic targets [54].

Based on this knowledge, our interest was focused on the protein expressions of the targets from such histone demethylases, namely methylated histone K27 (Figure 5B), which play a critical role in the regulation of developmental genes from cancer cells by stabilizing bivalent chromatin [55].

Downregulations to about 80% by simvastatin was observed for histone K27 in PC-3 cells, in MDA-MB-231, U-2 OS, and MG-63 cells the ibandronate-associated downregulation was about 50% (Figure 5B).

## 3. Discussion

In our previous studies, we demonstrated that statins and bisphosphonates suppress the one-carbon metabolism and induce epigenetic alterations in tumor cells [5]. Statins act through inhibition of 3-hydroxy-3-methylglutaryl-CoA reductase (HMGCR) by contrast to the bisphosphonates, which act several steps downstream in this pathway as inhibitors of farnesyltransferase. This leads to an accumulation of isopentenyl-pyrophosphate (IPP), which also acts as a genoprotective agent and thus could be responsible for a weaker effect on gene-regulation [56], as shown in Table 6.

Based on these results, in this study we studied the effect of such mevalonate pathway inhibitors on metabolic processes. It was a challenge to find out the background of drug-responses of cancer cell-types, which are characterized by remarkable differences not only in their growth rates but also in their epithelial or mesenchymal background. A key metabolite related to cell proliferation rate is NADPH, which is essential for the synthesis of dTMP and therefore for DNA synthesis and replication during cell division and replication. For synthesis of dTMP, it is generated by an enzymatic reaction involving three enzymes (thymidylate synthetase (TYMS), dihydrofolate reductase (DHFR), serine hydroxymethyltransferase 1 and 2 (SHMT1/2) where DHFR needs NADPH to provide 5,10-methylene-THF. Importantly, further NADPH producing reactions and pathways like glycolysis include the NADPH-producing pentose-phosphate cycle as well as the KREBS or tricarboxylic acid cycle [57,58].

The differences in NADP(H) levels measured in this study after treatment with simvastatin or ibandronate could be linked to a general downregulation of the energy metabolism. Indeed, ibandronate had just a minor effect on NADPH, which was significantly reduced by simvastatin in PC-3 and MDA-MB-231 cells (Figure 2A,B). Thus, we could not confirm a previous report indicating that simvastatin might protect MG-63 osteosarcoma cells from oxidative stress [59], which could be a result of an induction of the anti-apoptotic BCL2 apoptosis regulator by the runt related transcription factor 2 (RUNX2) in response to the treatment with hydrogen peroxide in that study [60]. As both genes had about the same basal expressions in the cell lines that were investigated in this study and were not significantly regulated, it rather appears possible that reduction of NADPH is a general sign for reduced energy metabolism in treated cells, also because data on the influence of simvastatin and ibandronate on the production of reactive oxygen species (ROS) appear contradictory (Supplementary Table S2 showing respective literature citations). Additionally, a comparative analysis of our transcriptomic data from Venn diagrams showed that simvastatin had a stronger impact on gene regulation than ibandronate in all investigated cell lines with the exception of MG-63 osteosarcoma cells (Table 6). However, it remains to be established if this is related to the (relatively) slow proliferation rate or to the osteogenic lineage of MG-63 cells or their different cell cycle response, which showed an arrest in the G2 phase.

Statins and bisphosphonates associated starvation affect mTOR-signaling resulting in an impaired uptake of nutrients such as essential amino acids including methionine [61,62], which is responsible for previously observed epigenetic alterations [5] and glucose metabolism [63,64,65]. This results in the stimulation of sestrin (SESN2) and inhibition of the mTOR [64,66,67] and RHOB pathway leading to stimulation of autophagy [68,69,70,71], as well as the downregulation of fatty acid synthase FASN [72]. The latter is partially associated with the upregulation of RHOB and sestrin [63,64,69] in mevalonate-dependent or independent manner.

Regarding prostate cancer, it has been postulated that ibandronate exerts its anti-proliferative effect through a reduction in the prenylation of RAC and via disruption of the NADPH oxidase complex [73]. The expression of RAC (gene name AKT1, not regulated in our study) at the protein level and the associated NADPH oxidases is cell type dependent and mirrors the mechanism of how bisphosphonates attenuate osteoclasts [74,75,76]. As simvastatin downregulates the DNA methyltransferase DNMT1 to a higher extent than the demethylating agent decitabine, a cell-line specific epigenetic reaction, which appears to be present in all investigated cell lines except U-2 OS, cannot be excluded [5].

The divergent responses induced by the tested drugs may lead to the hypothesis that the ectodermal (epithelial) origin of MDA-MB-231 and PC-3 cells versus the mesothelial origin of the osteosarcoma cell lines U2-OS and MG-63 might play a role in the observed differences. Furthermore, mutations in the retinoblastoma gene (RB1) as found in MDA-MB-231 and PC-3 cells [25,26] but not in MG-63 and U-2OS cells might also be partially responsible for the observed effects [77,78]. However, as the fast proliferating osteosarcoma cell line Saos-2 is more sensitive towards bisphosphonate-treatments [79] than U-2 OS or MG-63 cells, it appears that the critical parameter is just the slower growth rate of MG-63 and U-2 OS cell lines and not the mesothelial background.

Recently, it has been shown that the above-mentioned downregulation of energy metabolism could play a role in the maintenance of a stem-cell-like status, where an increased autophagy plays a decisive role [80]. Inhibitors of the mevalonate pathway are known to induce lysosomal activity and associated effects on autophagy [81].

SESN2, which is a key molecule of the autophagy pathway [36], was significantly upregulated in most of our treated cell lines (Figure 3). A concordant stimulation of the small GTPase *RHOB* could indicate an increase in protein degradation through an endolysosomal pathway [38], especially in simvastatin-treated PC-3 and MDA-MB-231 cells. SESN2 belongs to the highly conserved gene family, playing a key role in processes of adaptation to extreme climatic conditions in Antarctica [68]. Their primary function of SESN2 is to sensor lysine availability for further transport to mTOR via the GATOR complex that consists of a series of GTPases. SESN2 is upregulated upon stoppage of lysine import, as in situations of nutrient deprivation, starvation, or intoxication [67,82,83]. The u-regulation of SESN2 in statin- or ibandronate-treated cells regulates the activity of AMP-activated protein kinase (AMPK) [66,84,85] via liver kinase B (LKB1) mediated phosphorylation, thus promoting a status of quiescence [86,87,88,89]. Interestingly, in osteoblasts there is also a link between sestrin cell cycle attenuating activity of vitamin D (VD). In fact, VD induces the production of sestrins and thus leads to a cell cycle arrest [90].

This has been associated with metabolic processes that are turned down in starving or quiescent cells, which do not proliferate but appear to be protected against necrosis or apoptosis: it is upregulated by simvastatin and ibandronate and is known to interact with a complex that interacts with GTPases of the RAG family to promote mTORC1 translocation to the lysosomal surface named GATOR2 in an amino-acid-sensitive manner. Thus, it functions as a negative regulator of this pathway by preventing proper mTORC1 localization to the lysosome in response to essential amino acids [82] in all investigated cell lines. SESN2 attenuates the import of essential amino acids such as methionine by inhibiting the NPRL2 (nitrogen permease regulator like 2) gene, which is also responsible for the uptake of transcobalamin 2 (TCN2) and cobalamin (vitamin B12) [62]. This could be responsible for the downregulation of the one carbon metabolism (folate cycle) and associated inhibition of the thymidylate synthase and downregulation of epigenetic regulators such as DNA-methyl-transferases [5]. Data also indicate SESN2 protects cells from glucose starvation-induced necroptosis [64]. SESN2 regulation has been demonstrated to occur via TP53 dependent and independent mechanisms [91,92,93,94].

The SESN2 [95] gene is closely associated and deacetylated by the NAD–dependent histone deacetylase SIRT1 by a similar mechanism as described for the retinoblastoma gene RB1, which is known for its role in the transition from the G1 to the S-phase and mutated in PC-3 and MDA-MB-231 but not in MG-63 and U2-OS cell lines [96].

SESN2 cooperates with the hypoxia-inducible gene REDD1/RTP801 (gene name: *DDIT4*), which is part of a pathway, where mTOR inhibition is induced by hypoxia AMPK [97]. This *DDIT4* gene was significantly stimulated in U-2 OS cells both by ibandronate and simvastatin. Inductions of REDD1/RTP801 together with SESN2 by DNA damage are required for phosphorylation of the controlling 4E-BP1 (gene name: *EIF4EBP1*) elongation factor in situations of DNA damage [98].

This gene and its metabolic background is tightly regulated by microRNAs [46], which confers also to some microRNAs, where the extent of regulation is associated with cell type *MIR21* microRNA (Table 4) which is known to be RAS-activated [47]. Thus, a minus 3-fold ibandronate-induced downregulation in U-2 OS osteosarcoma cells might be associated with RAS-inactivation in this cell line.

As topoisomerase-inhibitors are known to act via the p-JUN-SESN2/AMPK pathway [99], the observed (both here and in a further study [100]) statin-mediated downregulation of topoisomerase (DNA) II α (TOP2A) could mimic this effect (see Table S1 in the supplementary materials).

Like *SESN2*, *ANLN* is also a Wnt/β-catenin responsive gene [101] and the relation of sestrin to Wnt/β-catenin and AMPK-signaling and histone deacetylase 5 is well documented [102].

The impact of ANLN on persistence of estrogen receptor positive breast cancer was shown by respective experiments showing a cell-cycle arrest in G2/M, lowered expression of cyclins D1, A2, and B1, as well as altered cell morphology [103].

Furthermore, an upregulation of *SESN2* (with concomitant downregulation of cell cycle regulators such as *DDIT4*, *CCNA2*, and *CCNB1*) and silencing of *ANLN* are known to facilitate but not necessarily induce apoptosis [104,105].

In addition, a downregulation of methyl-histones appears to be associated with the above-mentioned mechanisms of growth arrest and starvation. This induces a downregulation of developmental genes, which is also known to be a sign of quiescence [106]. This could also provide some explanation for the induction of growth arrest in cancer cells upon treatment with statins or bisphosphonates.

## 4. Materials and Methods

### 4.1. Cell Culture and NADP^+^/NADPH Analyses

Cell cultures and NADP/NADPH analyses were performed as previously described [5]. Media for cell cultures were from Sigma-Aldrich (DMEM-F12, St. Louis, MO, USA) or Biochrom (αMEM, Berlin, Germany).

### 4.2. Analysis of Gene Expression and Transcriptomics

Gene expression analyses were performed according to described protocols [5] with a quantitative real time PCR analysis system using a respective machine from Qiagen (Hilden, Germany), followed by evaluation using the comparative *C*_t_ method [107]. For transcriptomics analysis, RNA was analyzed on Affymetrix Arrays (Type Human Gene 2.0 ST Array, Thermo Fisher Scientific, Waltham, MA, USA) using the customized service from Kompetenzzentrum für Biofluoreszenz (Regensburg, Germany). The Pathvisio software [108] was used for detailed evaluation of signaling networks.

### 4.3. Flow Cytometry Analysis

Flow cytometric analysis for evaluation of cell cycle status was performed as previously described [109].

### 4.4.Protein Isolation and Immunoblotting

Whole cell protein extracts were prepared using SDS sample buffer (2% SDS, 100 mM β-mercaptoethanol, and 125 mM Tris-HCl, pH = 6.8) and heated at 95 °C for 5 min.

For immunoblotting analysis, 15 μg of protein extracts were separated on 10% SDS poly-acryl amide gels, transferred to nitrocellulose membranes (Millipore, Billerica, MA, USA), and blocked overnight with 10% blocking reagent (Roche, Basel, Switzerland) in 50 mM Tris buffered 125 mM NaCl solution (pH = 8.0). The following primary antibodies were used: Histone H3K27me2 antibody (pAb) activemotif no 39345; KDM7A (=JHDM1D) Antibody (PA5-25040, Thermo Fisher Scientific, Waltham, MA, USA), β-actin (ACTA1, A2066, Sigma-Aldrich, St. Louis, MO, USA), and histone H3 (D1H2, Cell Signaling Technology, Danvers, MA, USA).

Washing was performed with TN buffer containing 0.01% Tween Binding of the secondary antibody (anti-rabbit IgG/anti-mouse IgG horseradish peroxidase-coupled) (Santa Cruz, Dallas, TX, USA) diluted 1:10,000 in 10% blocking solution followed by detection with the BM chemo-luminescence immunoblotting kit (Roche, Basel, Switzerland), which was carried out as described by the supplier. Chemo-luminescence was measured with an image acquisition system (Vilber Lourmat, Marne-la-Vallée, France). Measurements are given as means of three immunoblots and representative blots are shown.

## 5. Conclusions

We would like to conclude with the hypothesis that statins and bisphosphonates may especially prevent the development and progression of fast-growing cancers by reducing nutrient uptake and energy metabolism. Thus, a reduction of tumor aggressiveness may be related to the effectiveness of statins and bisphosphonates in the downregulation of several metabolic pathways. However, it should be mentioned that these drugs appear to induce a (reversible) status of quiescence rather than cell death at least in about 30% of the cell population from respective cell lines. However, the overall efficacy of either type of drug is likely to be limited due to escape from inhibition or death of a significant proportion of the cell population.

## Figures and Tables

**Table 1:** Fold downregulation of DNA polymerase A1 (*POLA1*).

Cell-line	Simvastatin	Ibandronate
PC-3	−2.88	−3.15
MDA-MB-231	−2.28	−1.33
U-2 OS	−1.24	−1.70
MG-63	−1.13	−1.52

Results of transcriptomic analyses show downregulation of the DNA polymerase A1 (*POLA1*). Fold downregulation of, for example, −2.88 means a reduction of gene expression down to 35%, −3.15 to 32%, −2.28 down to 35%, and 1.70 to 59%. Regulations of +/− 1.5 are not significant.

**Table 2:** Fold downregulation of cyclin A2 (*CCNA2*).

CCNA2	Simvastatin	Ibandronate
PC-3	−12.71	−2.47
MDA-MB-231	−15.84	−1.05
U-2 OS	−1.16	−1.13
MG-63	−1.12	−1.03

Results of transcriptomic analyses show downregulation of the cell cycle regulator *CCNA2*. Fold downregulation of, for example, −12.71 means a reduction of gene expression down to 8%, −15 to 6.3%, and −2.47 down to 40%. Regulations of +/− 1.5 are considered as not significant.

**Table 3:** Fold downregulation of cyclin B1 *CCNB1*.

CCNB1	Simvastatin	Ibandronate
PC-3	−6.68	−1.87
MDA-MB-231	−9.2	−1.1
U-2 OS	−1.02	−1.62
MG-63	−1.2	−1.56

Results of transcriptomic analyses show downregulation of the cell cycle regulator *CCNB1*. Fold downregulation of, for example, −6.68 means a reduction of gene expression down to 15%, −9.2 to 11%. Regulations of +/− 1.5 are considered as not significant.

**Table 4:** Fold downregulation of forkhead box M1 *FOXM1*.

FOXM1	Simvastatin	Ibandronate
PC-3	−5.25	−2.43
MDA-MB-231	−4.00	1.07
U-2 OS	−1.14	−1.13
MG-63	−1.22	−1.02

Results of transcriptomic analyses show downregulation of the G2/M phase regulator *FOXM1*. Fold downregulation of, for example, −5.25 means a reduction of gene expression down to 19%, −2.43 to 41%, and −4 to 25%. Regulations of +/− 1.5 are not significant.

**Table 5:** Drug-induced increase of NADP^+^ (in relation to NADPH) is associated with doubling time.

Cell Line	Used Conc. (EC50)	Simvastatin NADP+/NADPH Vs. Co	Doubling Time Citation
PC-3	1 µM	1.54	13.2 h [27]
MDA-MB-231	0.5 µM	2.42	24 h [28]
U-2 OS	3 µM	2.43	28 h [29]
MG-63	10 µM	4.56	38 h [30]
		**Ibandronate**	
PC-3	50 µM	1.07	13.2 h [27]
MDA-MB-231	50 µM	0.95	24 h [28]
U-2 OS	50 µM	2.71	28 h [29]
MG-63	50 µM	3.57	38 h [30]

**Table 6:** Drug-induced gene regulation, based on evaluation of respective gene-chips.

Cell Line—Regulation	Simvastatin	Ibandronate	Overlap
MDA—upregulation	516	35	4
MDA—downregulation	1450	60	26
MG63—upregulation	81	278	37
MG63—downregulation	252	574	123
PC3—upregulation	572	290	216
PC3—downregulation	637	334	228
U2OS—upregulation	322	320	78
U2OS—downregulation	74	175	21

Results of transcriptomic Venn-diagram analyses showing the number of genes that were either regulated with simvastatin or by ibandronate or by both drugs, which is termed as “overlap”.

**Table 7:** Fold regulation of NADPH-oxidase *NOX4*.

NOX4	Simvastatin	Ibandronate
PC-3	1.53	1.22
MDA-MB-231	1.52	−1.07
U-2 OS	−3.22	1.83
MG-63	−1.29	1.11

Results of transcriptomic analyses show downregulation of the NADPH-oxidase *NOX4*. Fold downregulation of, for example, −3.22 means a reduction of gene expression down to 31%, −1.29 to 77%.

**Table 8:** Fold regulation of nitric oxide synthase *NOS1*.

NOS1	Simvastatin	Ibandronate
PC-3	−1.18	1.22
MDA-MB-231	1.08	−1.07
U-2 OS	3.56	1.83
MG-63	1.19	1.11

Results of transcriptomic analyses shows upregulation of the endothelial nitric oxide synthase *NOS1* in U-2 OS cells. Regulations of +/− 1.5 are not significant.

**Table 9:** Fold downregulation of prenyl (decaprenyl) diphosphate synthase subunit 1 *PDSS1*.

PDSS1	Simvastatin	Ibandronate
PC-3	−2.43	−2.65
MDA-MB-231	−2.61	−1.03
U-2 OS	−1.31	1.00
MG-63	−1.07	−1.15

Results of transcriptomic analyses show downregulation of the Prenyl (decaprenyl) diphosphate synthase subunit 1 *PDSS1*. Fold downregulation of, for example, −2.43 means a reduction of gene expression down to 41%, −2.65 or −2.61 to 38%.

**Table 10:** Fold upregulation of *MIR21*.

MIR21	Simvastatin	Ibandronate
PC-3	1.3	−1.2
MDA-MB-231	1.1	1.0
U-2 OS	1.0	3.2
MG-63	1.1	1.0

Results of transcriptomic analyses shows upregulation of the *MIR21* in U-2 OS cells. Regulations of +/−1.5 are not significant.

**Table 11:** Fold downregulation of the histone methylase *EZH2*.

EZH2	Simvastatin	Ibandronate
PC-3	−1.9	−1.3
MDA-MB-231	−2.2	−1.6
U-2 OS	−1.2	−1.1
MG-63	−1.2	−1.1

Results of transcriptomic analyses show downregulation of the histone methylase *EZH2*. Fold downregulation of, for example, −2 means a reduction of gene expression down to 50%. Regulations of +/−1.5 are not significant.

**Table 12:** Fold upregulation of histone demethylase *KDM7A*.

KDM7A	Simvastatin	Ibandronate
PC-3	5.6	3.8
MDA-MB-231	2.0	1.0
U-2 OS	3.2	4.0
MG-63	1.5	1.7

Results of transcriptomic analyses show upregulation of the histone methylase *KDM7A*. Fold downregulation of, for example, 2 means an increase of gene expression up to 200%. Regulations of +/−1.5 are not significant.

## Supplementary Materials

Supplementary materials can be found at www.mdpi.com/1422-0067/18/9/1982/s1.
