# Supplementary material for: Statin and Bisphosphonate Induce Starvation in Fast-Growing Cancer Cell Lines
Source: Int J Mol Sci. 2017 Sep 15;18(9):1982. doi: 10.3390/ijms18091982 (PMC5618631; doi:10.3390/ijms18091982)
Supplement: Supplementary file 1 [file ijms-18-01982-s001.pdf]

Supplementary Table S1:

Amount of "Pubmed"-results with the 7 best-regulated genes (July 2017)

Gene	"Cell cycle" AND "gene"
POLA1	10
RHOB	120
TOP2A	86
TYMS	31
ANLN	10
SESN2	24
DDIT4	46
CCNA2	570
CCNB1	3446

Supplementary Table 2:
Anti-oxidant versus Pro-oxidant reports on regulators of mevalonate metabolism

A	**Statins**	
	**Statins Antiox**	**Statins Pro Ox = Pro ROS**
1	Normal podocytes PMID: 25240415	A549 lung cancer cells (Sim upregulates SOD2) PMID: 25158572
2	Cardiovasc system PMID: 25218292	Mouse (feeding experiment) PMID: 25106875
3	Renal and alveolar epithelial cells PMID: 25207459	Malignant mesothelioma and lung cancer cells PMID: 25096993
4	Cardiovasc system PMID: 25132378	Rat hepatocytes PMID: 24754008
5	Human B lymphoma cell PMID: 24518247	In Melanoma by activating P53/P21 pathways PMID: 23933099
6	Serum (human) PMID: 24401239	Rat hepatocytes PMID: 23761184
7	Human endothelial cells PMID: 24375611	OCM-1 (choroidal melanoma) cells (PMID: 23727160
8	Serum (human) PMID: 24263084	A549 Lung cancer cells PMID: 23661227
9	Skin (human) PMID: 24155025	Human lymphoma cells PMID: 23449454
10	Immune system (human) PMID: 24121054	Myeloma PMID: 21958871
11	Platelets (human) PMID: 23992095	Lung cancer PMID: 23045963
12	Cardiovasc system PMID: 23948511	MCF-7 breast cancer PMID: 18608208
13	Brain endothel (nucleus tractus solitarii) PMID: 23889671	malignant pheochromocytomas and paragangliomasPMID: 24846270
14	Cardiovasc system PMID: 23643589	Human monocytes and macrophages PMID: 23159926
15	Cardiovasc system PMID: 23608227	Microvessels (swine) PMID: 18179927
16	Human endothelial cells PMID: 23608189	Myocardial tissue (swine) PMID: 18598320
17	Mouse: high fat diet induced stress PMID: 23567805	Human peripheral blood leukocytes PMID: 18161561
18	Smooth muscle PMID: 23541442	Human peripheral blood leukocytes PMID: 18485436
19	BrainPMID: 23500607	Atorvastatin (ATO) exposure (100µM) for 24h increased ROS levels, reduced the percentage of live myeloblast cells PMID: 25769432
20	Human endothelial cells PMID: 23426387	link between statin-induced mitochondrial oxidative stress and activation of the mitochondrial apoptosis signaling pathway in glycolytic skeletal muscle, PMID: 26414931
21	Mouse endothelial cells PMID: 23259560	Myopathy, characterized by mitochondrial oxidative stress, occurs in

		approximately 10 % of statin-treated patients. PMID: 27245589
22	Human macrophages PMID: 23141585	Statin increased hepatic reactive oxygen species (ROS) PMID: 27428373
23	Human endothelial cells PMID: 23030449	Statin-induced myotoxicity by ROS PMID: 28223230
24	Human platelets PMID: 23022230	atorvastatin (AS) can increase intracellular reactive oxygen species (ROS) and induce necrotic cell death autophagy in NIT-1 cells.<br>PMID: 28004006
25	Cardiovasc system PMID: 23016719 [	Mitochondrial oxidative stress caused by statin treatment also signals for cellular antioxidant system responses such as catalase upregulation. These results suggest that the detrimental effects of statins on muscle mitochondria could be prevented by co-administration of a safe antioxidant such as creatine or CoQ10.<br>PMID: 28424622
26	Retinal pigment epithelium (RPE) PMID: 22918643	Atorvastatin enhanced the cell killing effect of irradiation by reducing endogenous ROS levels and prolonging the lifespan of radiation-induced ROS via a decrease in the level of NOXs and SOD activity.<br>PMID: 28260074
27	Cardiovasc system PMID: 22862793	Administration of simvastatin was followed by a decrease in activities of superoxide dismutase and cytochrome oxidase in rat mitochondria, which attested to dysfunction of the respiratory chain. PMID: 28239789
28	Injured lung tissue PMID: 22701728	
29	Muscle (rabbit) PMID: 22661508	
30	Cardiovasc system PMID: 22652672	
31	Cardiovasc system PMID: 22405985	
32	Cardiovasc system PMID: 22394340	
33	Human Kidney PMID: 22386936	
34	Cochlear tissue (mouse ear) PMID: 22366511	
35	Cardiomyocytes PMID: 22365145	
36	Cardiovasc system PMID: 22293859	
37	Diabetic Polyneuropathy PMID: 25214797	
38	Arsenic-induced hypertension PMID: 25218292	
39	Reduction of the proadhesive and prothrombotic endothelial cell phenotype	

	induced by cocaine and plasma from cocaine consumers PMID: 25234816	
40	The antitumor activity of LCL-SIM (liposomal simvastatin) depends on the presence of functional tumor-associated macrophages (TAM) in tumor tissue and is mainly based on the reduction of the TAM-mediated oxidative stress. PMID: 25444912	
41	Protective effect on cardiovascular disease through the inhibition of SIRT1 expression in coronary artery disease patients. PMID: 25582759	
42	Statin decreases the oxidative stress caused by noise exposure PMID: 25599753	
43	Simvastatin and rosuvastatin significantly ameliorate experimental colitis in rats PMID: 25821305	
44	Antioxidant activity of statins appear to be the main mechanisms involved in reducing the frequency of mutant spots and consequent modulation of the damage induced by Doxorubicin. PMID: 25846503	
45	Simvastatin clearly lowers the serum levels of CRP and IL-6, and the white blood cell count in dialysis patients. PMID: 25866715	
46	Anti epilepsy PMID: 25901524	
47	Neuroprotective PMID: 25912435	
48	Anti Osteoarthritis PMID: 25933963	
49	Anti perioperative myocardial damage PMID: 25968407	
50	Regulates Coenzyme Q10 a naturally-occurring coenzyme with antioxidant effects	
51	Against antioxidant/antiinflammatory properties of HDLs in hypercholesterolemics PMID: 26084126	
52	Anti-inflammatory; Antioxidant; against Biphosphonate; Gastric damage PMID: 26403426	
53	Antioxidant; Arylesterase; Cardiovascular disease; PMID: 26416579	
54	Ameliorative effect of statin therapy on oxidative damage in heart tissue	

	PMID: 26456720	
55	Simvastatin inhibits oxidative stress via the activation of nuclear factor erythroid 2-related factor 2 signaling in trophoblast cells. PMID: 26556031	
56	Fluvastatin Decreases Oxidative Stress in Kidney Transplant Patients PMID: 26707305	
57	Antioxidation Effect of Simvastatin in Aorta and Hippocampus PMID: 26798426	
58	modulating effect of simvastatin was also observed on DNA damage induced by DXR (doxorubicin) PMID: 26829615	
59	Antagonistic effect and mechanism of Rosuvastatin on myocardial apoptosis<br>PMID: 26849935	
60	non-cholesterol-lowering actions (anti-inflammatory, antioxidant and antithrombotic)<br>PMID:26930419	
61	antioxidative properties of atorvastatin through down-regulating NOX1<br>PMID: 26957227	
62	atorvastatin and artichoke leaf tincture reduce oxidative stress<br>PMID: 27071286	
63	Antioxidant activity of simvastatin prevents ifosfamide-induced nephrotoxicity.<br>PMID: 27087071	
64	simvastatin-conferred protection of eNOS (endothelial nitric oxide synthase) activation, NO production, and angiogenesis as well as the clinical outcome of cardiovascular events.<br>PMID: 27091343	
65	effect of each statin on the antioxidant and cytoprotective enzyme, heme-oxygenase 1. PMID: 27207105	
66	Statin use decreased the IL-1β and MPO levels PMID: 27290718	
67	Simvastatin induces the activation and nuclear translocation of Nrf2 and the expression of various anti-oxidant enzymes via ERK and PI3K/Akt	

	pathway PMID: 27323826	
68	protective effects of simvastatin against alendronate-induced gastric ulceration. PMID: 26403426	
69	Simvastatin protects human osteosarcoma cells (MG63)from oxidative stress-induced apoptosis through mitochondrial-mediated signaling. PMID: 22012179	
70	Simvastatin prevents ifosfamide-induced nephrotoxicity. PMID: 27087071	
71	effects of statins are anti-inflammatory, anti-proliferative, antioxidant. PMID: 27146293	
72	Simvastatin-induced anti-oxidant enzymes. PMID: 27323826	
73	Treatment with the cholesterol-lowering drug lovastatin reduced cell death by inhibiting the production of reactive oxygen species, but did not prevent lysosomal cholesterol increase. PMID: 28109635	
74	Pravastatin (PS) does not cause ROS and cell death but also induces autophagy. PMID: 28004006	
75	Atorvastatin reduced ROS. PMID: 27649495	
76	Atorvastatin and rosuvastatin have similar effects on oxidative status in patients with acute myocardial infarction PMID: 28429691	
77	Antioxidative potentials of atorvastatin and simvastatin during the different degrees of hyperhomocysteinemia (HHcy) in rats PMID: 28620818	

B	Bisphosphonates	
	**Bisphosphonates Pro Antiox**	**Bisphosphonates Contra Antiox = Pro ROS**
1	Human endothelial cells PMID: 16440584	Prostate cancer and multiple myeloma<br>Pro-oxidant synergism of Panbinostat (HDACi) and Bisphosphonate<br>PMID: 24157872
2	Human endothelial cells PMID: 16216433	Fibrosarcoma PMID: 23242142
3	Cells (hematopoietic and musculuskeletal) infected with Paramyxovirus in Paget's disease PMID: 10321921	Human erythrocytes PMID: 21843812
4	Bisphosphonates alleviated oxidative stress induced by cancer.<br>PMID: 24308847	Lung cancer PMID: 23045963
5	Alendronate anti ROS in osteoblasts (2014, Lezcano V et al)<br>PMID: 24698731	ZA may aggravate renal injury during I/R by increasing cytokine production and apoptosis. PMID: 25746831
6	reducing nitric oxide synthase expression<br>PMID: 24831353	Bisphosphonate-related osteonecrosis of the jaw PMID: 26026888
7	increased oxidative stress induced by periodontal infection in rats can be ameliorated by bone-targeted antiresorptives. PMID: 25101489	Alendronate-induced gastric ulceration.<br>PMID: 26403426
8	3-keto-1,5-bisphosphonates 1 could be considered as safe antioxidant agents PMID: 25483977	Use of Zoledronic Acid Augments Ursolic Acid-Induced Apoptosis in Human Osteosarcoma Cells through Enhanced Oxidative Stress and Autophagy.<br>PMID: 27916903
9	Ibandronate exerted cellular antioxidant effects through the vasoprotective effects on the impaired endothelium<br>PMID: 27035426	
